# Pseudocapsule status combined with pathological parameters predicts prognosis in renal cell carcinoma

**DOI:** 10.3389/fonc.2026.1779539

**Published:** 2026-06-22

**Authors:** Jiaxi Yao, Wei Xi, Hong Wang, Yiru Hou, Feifei Cui, Xi Chen, Huiling Sun, Ruirui Ma, Jianming Guo, Xiaoyi Hu

**Affiliations:** 1 Department of Urology, Hexi University Affiliated Zhangye People’s Hospital, Zhangye, China; 2 Department of Urology, Zhongshan Hospital, Fudan University, Shanghai, China; 3 Department of Anesthesiology and Perioperative Medicine, Zhangye Second People’s Hospital, Zhangye, China; 4 Medical College of Hexi University, Zhangye, China

**Keywords:** overall survival, pathological parameters, prognosis, pseudocapsule, renal cell carcinoma

## Abstract

**Background:**

We evaluated whether pseudocapsule status, alone and in combination with pathological parameters, could serve as a prognostic marker in human renal cell carcinoma (RCC).

**Methods:**

We retrospectively analyzed 1560 patients with RCC who underwent surgery at a single institution between 2007 and 2012. The patients were randomly assigned to training (n=780) and validation (n=780) cohorts. Pseudocapsule status was classified as grade 0 (intact pseudocapsule without breakthrough), grade 1 (pseudocapsule invasion), and grade 2 (pseudocapsule breakthrough or absence). A pseudocapsule-pathological parameter score (PPS) was constructed by integrating pseudocapsule status with pathological type, Fuhrman grade, nuclear necrosis, sarcomatoid transformation, microvascular infiltration, Eastern Cooperative Oncology Group performance status score, and tumor-node-metastasis stage. The scores were categorized as low, intermediate, and high. Overall survival and relapse-free survival were evaluated using Kaplan–Meier and Cox regression analyses.

**Results:**

Among the 1560 patients, 529 had grade 0 pseudocapsules, 701 had grade 1 pseudocapsules, and 330 had grade 2 pseudocapsules. The Kaplan–Meier curves revealed better overall survival in patients with grade 0 pseudocapsules than those with grades 1 and 2. Multivariate analyses identified grade 2 pseudocapsule status as an independent predictor of worse overall survival (hazard ratio 3.94, 95% confidence interval 2.09–7.43, *p* < 0.001) and relapse-free survival (hazard ratio 2.87, 95% confidence interval 1.72–4.81, *p* < 0.001) in the training cohort. The validation cohort showed similar findings (overall survival: hazard ratio 2.64, 95% confidence interval 1.55–4.49, *p* < 0.001; relapse-free survival: hazard ratio 2.92, 95% confidence interval 1.81–4.71, *p* < 0.001). In addition, the PPS was independently associated with overall survival. Compared with the low PPS group, the high PPS group showed significantly worse prognosis in both cohorts (training: hazard ratio 33.58, 95% confidence interval 15.52–72.67, *p* < 0.001; validation: hazard ratio 18.10, 95% confidence interval 9.35–35.03, *p* < 0.001).

**Conclusions:**

Pseudocapsule status demonstrated good prognostic implications in RCC. Furthermore, the new PPS system, which combines pseudocapsule status with established pathological parameters, may better predict patient prognosis.

## 1 Introduction

Renal cell carcinoma (RCC) is a common malignancy of the urinary system (1), with an estimated 81, 610 new cases and 14, 390 deaths in the United States in 2024 (2). In China, the incidence of RCC is 4.02 per 100000, and both its incidence and mortality rates are rising (3). Partial nephrectomy remains the preferred surgical treatment for patients with early-stage RCC (4). The primary objective of partial nephrectomy is complete tumor excision, which often requires resection along the tumor pseudocapsule or with a portion of normal kidney tissue (5).

The formation of a pseudocapsule is closely related to tumor growth and the response of the surrounding tissues. Tumor progression may trigger an inflammatory response that causes fibrosis in the surrounding tissues, forming a more distinct boundary and thus creating the pseudocapsule, which is clearly visible in pathological sections. Pseudocapsules are a common phenomenon in solid-organ tumors. The pseudocapsule separates the renal tumor from the surrounding normal kidney tissue through fibrous compression, thereby providing a natural anatomical plane for surgery (6). Although the presence of a pseudocapsule is critical for guiding surgery, few studies have specifically investigated the relationship between the pseudocapsule and patient prognosis.

Some studies suggest that pseudocapsule invasion is unrelated to patient prognosis, while others report that pseudocapsule invasion is a poor prognostic factor. In addition, current prognostic models for RCC have some limitations. The pseudocapsule, as an important component of renal tumors, plays a significant role during surgery. Although previous studies have suggested that the morphological characteristics of the pseudocapsule can predict the prognosis of renal cancer patients, it has not received sufficient attention in routine pathological assessment. Fibrosis at the tumor margin is one of the important prognostic indicators for liver cancer (7). Similarly, pseudocapsule status may provide clinically relevant prognostic information in RCC. For localized RCC, the University of California Integrated Staging System (UISS) and Stage, Size, Grade, and Necrosis (SSIGN) scores are standard prognostic indicators; however, these do not consider pseudocapsule invasion (8).

Given these considerations, we hypothesized that incorporating pseudocapsule status into prognostic assessment and combining it with established pathological parameters could improve prognostic prediction in patients with RCC. Therefore, we evaluated whether pseudocapsule invasion is an independent predictor of overall survival (OS) and relapse-free survival (RFS), and whether combining pseudocapsule status with pathological parameters improves prognostic performance compared with the SSIGN score.

## 2 Materials and methods

### 2.1 Study population

We reviewed the records of 1560 patients who underwent radical or partial nephrectomy at Zhongshan Hospital, Fudan University, between 2007 and 2012. The patients were randomly divided into training and validation cohorts, with 780 patients in each cohort. A 1:1 ratio was used to ensure adequate sample size and event distribution in both cohorts for model development and validation. All surgical specimens were pathologically confirmed as RCC. Tumor staging was determined based on the American Joint Cancer Committee tumor-node-metastasis (TNM) staging system, and tumor grading was assessed using the International Society of Urological Pathology (ISUP) grading systems.

Ethical ratification was authorized by the Clinical Research Ethics Committee of Zhongshan Hospital, Fudan University (Shanghai, China) with the approval number B2016-161R. Verbal informed consent was obtained from each patient.

### 2.2 Histopathological assessment of pseudocapsule status

Pseudocapsule status was evaluated on hematoxylin and eosin (H&E)-stained whole-mount sections at the tumor–parenchyma interface. Grade 0 (intact pseudocapsule) was defined as a continuous, uninterrupted fibrous layer separating the tumor from adjacent renal parenchyma, with no tumor cells reaching or penetrating the capsular boundary. Grade 1 (pseudocapsule invasion) was defined as tumor cell extension into the fibrous layer, characterized by either focal infiltration (involving <25% of the capsular circumference) or diffuse involvement (≥25%), but without complete transection of the fibrous layer or contact with perinephric/perisinusoidal fat. Grade 2 (pseudocapsule breakthrough or absence) was defined as (i) complete disruption of the fibrous layer, with tumor cells directly abutting or extending into peritumoral adipose tissue, or (ii) the absence of a discernible pseudocapsule at the tumor–parenchyma interface.

Borderline or ambiguous cases (e.g., artifactual capsular tearing, tangential cutting, or marked inflammation obscuring the interface) were reviewed jointly by two pathologists in a blinded manner. When necessary, deeper sections or adjacent tissue blocks were re-evaluated, and any remaining discrepancies were resolved by consensus with a third senior pathologist.

The primary endpoints were OS and RFS, and this study has been reported in accordance with the Transparent Reporting of a Multivariable Prediction Model for Individual Prognosis or Diagnosis guidelines (9).

### 2.3 Statistical analysis

Continuous variables were tested for normality using the Shapiro–Wilk test. Normally distributed variables were compared using the independent samples *t*-test, whereas non-normally distributed variables were compared using the Mann–Whitney U test. Categorical variables were compared using the chi-square test or Fisher’s exact test, as appropriate. Baseline characteristics were compared between the training and validation cohorts to ensure balance.

OS and RFS were defined as the time from the date of surgery to the date of death or relapse, respectively, with censoring at the last follow-up. Survival curves were estimated using the Kaplan–Meier method and compared using the log-rank test.

The pseudocapsule-pathological parameter score (PPS) was constructed based on the regression coefficients derived from the final multivariate model. Receiver operating characteristic (ROC) curve analysis was performed to evaluate the predictive accuracy of the PPS system for 5-year OS, and the area under the curve (AUC) was calculated. The AUC of the PPS system was compared with those of the SSIGN score and TNM stage using the DeLong test.

All analyses were conducted with SPSS version 21.0 (IBM) and GraphPad Prism 6 (GraphPad Software Inc). *P* < 0.05 (two-sided) was considered statistically significant.

## 3 Results

### 3.1 General patient information and prognostic significance of the pseudocapsule status

**Figure 1:**
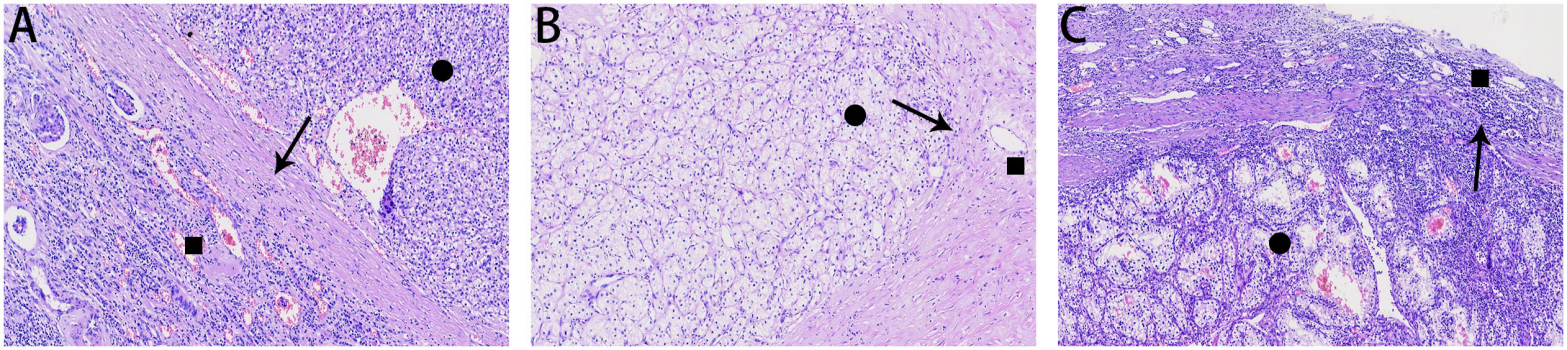
Representative histopathological features of pseudocapsule status. **(A)** Grade 0, intact pseudocapsule without tumor breakthrough. **(B)** Grade 1, tumor invasion into the pseudocapsule. **(C)** Grade 2, tumor breakthrough of the pseudocapsule. Black arrow: pseudocapsule; black circle: tumor area; black square: normal tissue area. Original magnification: ×100.

The clinical and pathological parameters did not differ significantly between the training and validation cohorts (**Table 1**). Among the 1560 included patients, 1077 were male and 483 were female. Histological subtypes included 1233 cases of clear cell renal cell carcinoma (ccRCC). Pseudocapsule status was classified as grade 0 in 529 patients, grade 1 in 701 patients, and grade 2 in 330 patients (**Figure 1**). The median follow-up duration for the entire cohort was 70 months.

**Table 1:** Clinical and pathologic characteristics of patients with renal cell carcinoma in the training and validation cohorts.

Characteristics	Total	Training cohort	%	Validation cohort	%	*p*-value
No. of patients	1560	780		780		
Sex						0.70
Male	1077	535	68.6	542	69.5	
Female	483	245	31.4	238	30.5	
Age, IQR = 56 (47–63)						0.76
≤56 years	824	415	53.2	409	52.4	
>56 years	736	365	46.8	371	47.6	
Pseudocapsule						0.44
Grade 0	529	274	35.1	255	32.7	
Grade 1	701	350	44.9	351	45.0	
Grade 2	330	156	20.0	174	22.3	
Tumor necrosis						0.85
Absent	1249	623	79.9	626	80.3	
Present	311	157	20.1	154	19.7	
Sarcomatoid transformation						0.17
Absent	1504	747	95.8	757	97.1	
Present	56	33	4.2	23	2.9	
MI						0.36
Absent	1319	666	85.4	653	83.7	
Present	241	114	14.6	127	16.3	
ECOG						0.38
0	1173	579	74.2	594	76.2	
1	387	201	25.8	186	23.8	
Histologic subtype						0.29
Clear cell	1233	608	77.9	625	80.1	
Non-clear cell	327	172	22.1	155	19.9	
Tumor nuclear grade						0.37
1	232	114	17.6	118	15.1	
2	768	370	47.4	398	51.0	
3	363	189	24.2	174	22.3	
4	197	107	13.8	90	11.6	
Initial TNM stage						0.26
Stage I	978	493	63.2	485	62.2	
Stage II	154	67	8.6	87	11.2	
Stage III	352	185	23.7	167	21.4	
Stage IV	76	35	4.5	41	5.2	
Tumor size median						0.88
≤4 cm	770	390	50.0	380	48.7	
>4 and ≤7 cm	522	257	32.9	265	34.0	
>7 cm	268	133	17.1	135	17.3	
Adjuvant immunotherapy						0.55
Absent	1067	529	67.8	538	69.0	
Present	493	251	32.2	242	31.0	
Adjuvant TKIs						0.18
Absent	1513	761	97.6	752	96.4	
Present	47	19	2.4	28	3.6	
Score						0.89
Low	455	231	29.6	224	28.7	
Intermediate	695	343	44.0	352	45.1	
High	410	206	26.4	204	26.2	

ECOG, Eastern Cooperative Oncology Group; grade 0, pseudocapsule intact; grade 1, pseudocapsule invasion; grade 2, pseudocapsule breakthrough; IQR, interquartile range; TKI, tyrosine kinase inhibitor; TNM, tumor-node-metastasis; MI, microvascular infiltration.

The training cohort included 274 (35.1%), 350 (44.9%), and 156 (20.0%) patients with pseudocapsule grades 0, 1, and 2, respectively. Similarly, the validation cohort comprised 255 (32.7%), 351 (45.0%), and 174 (22.3%) patients, respectively.

**Figure 2:**
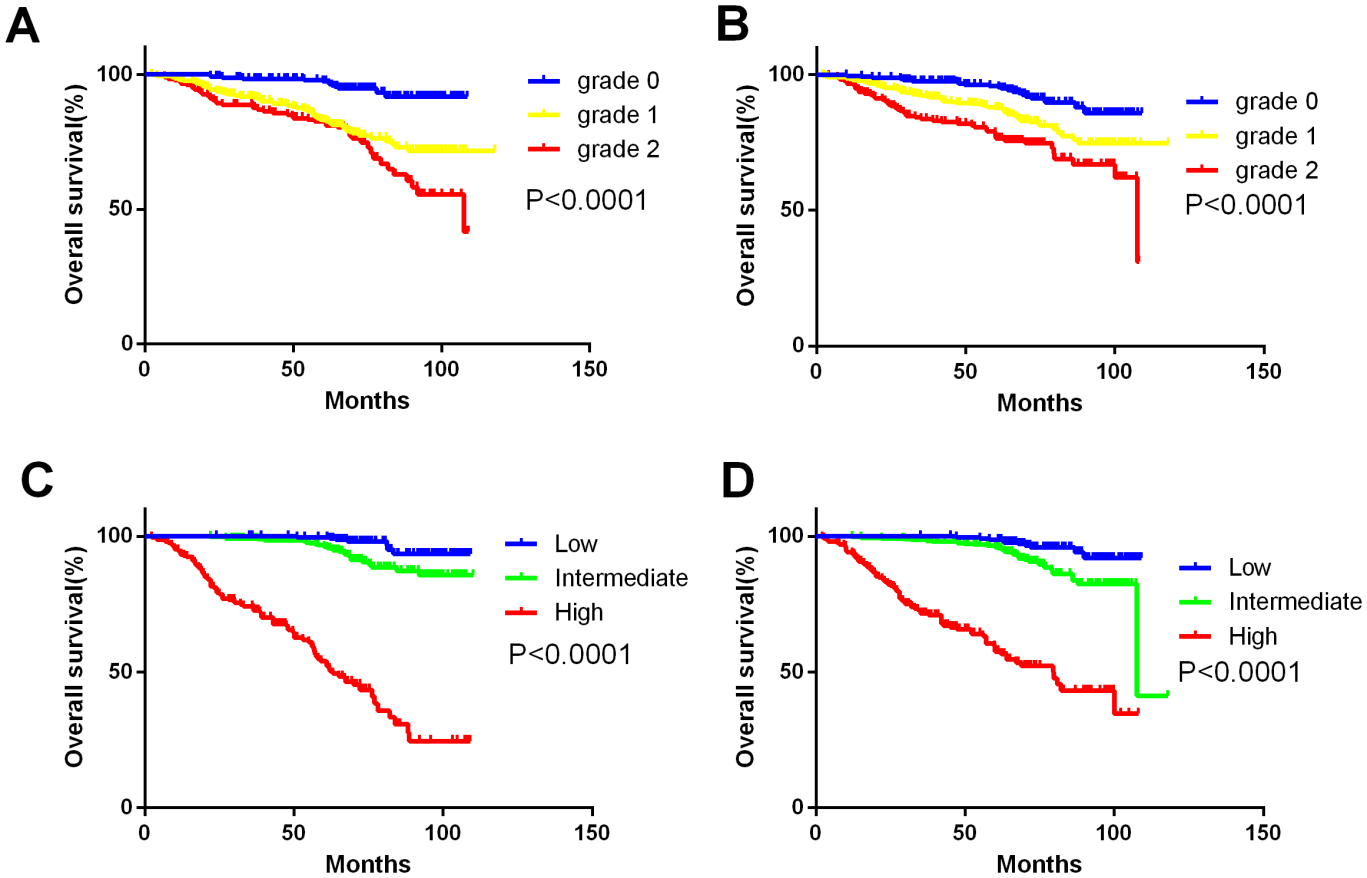
Kaplan-Meier curves according to pseudocapsule state and the pseudocapsule-pathological parameter score (PPS) in the training and validation cohorts. Kaplan–Meier analysis of overall survival (OS) according to pseudocapsule status in the **(A)** training cohort and **(B)** validation cohort. Kaplan–Meier analysis of OS according to the PPS system in the **(C)** training cohort and **(D)** validation cohort. *p*-value was calculated by log-rank test.

OS was closely associated with pseudocapsule status. Kaplan–Meier curve analysis showed that patients with grade 2 pseudocapsules had the worst survival prognosis (*p* < 0.001) in the training cohort, with a median OS of 107.6 months. Similar results were observed in the validation cohort (**Figures 2A, B**).

### 3.2 Univariable and multivariable analyses of prognostic factors

Variables with *p* < 0.05 in the univariable analyses were entered into the multivariable Cox regression model.

For OS in the training cohort (**Table 2**), age >56 years was independently associated with worse OS (hazard ratio [HR] 1.69, 95% confidence interval [CI] 1.18–2.41, *p* = 0.004). Compared with grade 0, grade 2 pseudocapsules were associated with a significantly higher risk of death (HR 3.94, 95% CI 2.09–7.43, *p* < 0.001), as were grade 1 pseudocapsules (HR 3.59, 95% CI 2.01–6.39, *p* < 0.001). Other independent predictors of OS included tumor nuclear grade (overall *p* < 0.001), initial TNM stage (overall *p* < 0.001), tumor necrosis (HR 1.83, 95% CI 1.23–2.73, *p* = 0.003), sarcomatoid transformation (HR 2.86, 95% CI 1.47–5.55, *p* = 0.002), microvascular infiltration (HR 2.21, 95% CI 1.43–3.41, *p* < 0.001), and Eastern Cooperative Oncology Group (ECOG) performance status score 1 vs 0 (HR 2.27, 95% CI 1.54–3.34, *p* < 0.001).

**Table 2:** Multivariable Cox regression analyses of factors associated with overall survival and relapse-free survival in the training cohort (n=780).

Variables	OS		RFS	
	HR (95% CI)	*p*-value	HR (95% CI)	*p*-value
Age				
>56 years *vs ≤*56 years	1.69 (1.18–2.41)	**0.004**	1.19 (0.86–1.65)	0.30
Histologic subtype				
Non-clear cell *vs* clear cell type	1.08 (0.69–1.66)	0.74	0.95 (0.63–1.43)	0.81
Tumor nuclear grade				
1		**<0.001**		**<0.001**
2	2.01 (0.90–4.48)		2.46 (1.05–5.75)	
3	3.29 (1.45–7.51)		3.92 (1.65–9.30)	
4	6.50 (2.72–15.52)		6.71 (2.72–16.56)	
Initial TNM stage				
I		**<0.001**		**<0.001**
II	2.42 (1.16–5.02)		1.89 (0.98–3.65)	
III	2.52 (1.63–3.91)		2.09 (1.40–3.13)	
IV	6.06 (3.31–11.09)		4.48 (2.48–8.11)	
Tumor size				
<4 cm		0.23		**0.01**
4–7 cm	1.40 (0.90–2.19)		1.86 (1.22–2.85)	
>7cm	1.53 (0.89–2.63)		1.98 (1.17–3.36)	
Pseudocapsule				
Grade 0		**<0.001**		**<0.001**
Grade 1	3.59 (2.01–6.39)		2.28 (1.43–3.64)	
Grade 2	3.94 (2.09–7.43)		2.87 (1.72–4.81)	
Tumor necrosis				
Present vs Absent	1.83 (1.23–2.73)	**0.003**	2.19 (1.52–3.12)	**<0.001**
Sarcomatoid transformation				
Present vs Absent	2.86 (1.47–5.54)	**0.002**	2.83 (1.61–4.97)	**<0.001**
MI				
Present vs Absent	2.21 (1.43–3.41)	**<0.001**	1.73 (1.15–2.62)	**0.01**
ECOG				
1 *vs* 0	2.27 (1.54–3.34)	**<0.001**	1.80 (1.25–2.59)	**0.001**
PPS				
Low		**<0.001**		**<0.001**
Intermediate	3.22 (1.42–7.32)		2.99 (1.44–6.19)	
High	33.58 (15.52–72.67)		27.32 (13.76–54.23)	

CI, confidence interval; ECOG, Eastern Cooperative Oncology Group; OS, overall survival; RFS, relapse free survival; TNM, tumor-node-metastasis; MI, microvascular infiltration; PPS, pseudocapsule-pathological parameter score. The bold p-values indicate a value less than 0.05, indicating a statistically significant difference.

Similar findings were observed in the validation cohort (**Table 3**). Independent predictors of OS included tumor nuclear grade (HR 2.98, 95% CI 1.41–6.29, *p* < 0.001), initial TNM stage (HR 6.58, 95% CI 3.66–11.83, *p* < 0.001), and tumor size (HR 2.42, 95% CI 1.40–4.18, *p* = 0.004). Grade 2 pseudocapsules were associated with a significantly higher risk of death compared with grade 0 pseudocapsules (HR 2.64, 95% CI 1.55–4.49, *p* < 0.001), as were grade 1 pseudocapsules (HR 1.79, 95% CI 1.07–2.99, *p* < 0.001), nuclear necrosis (HR 1.79, 95% CI 1.23–2.92, *p* = 0.02), sarcomatoid transformation (HR 2.72, 95% CI 1.45–5.08, *p* = 0.002), microvascular infiltration (HR 1.89, 95% CI 1.23–2.92, *p* = 0.004), and ECOG performance status (HR 2.14, 95% CI 1.42–3.21, *p* < 0.001).

**Table 3:** Multivariable Cox regression analyses of factors associated with overall survival and relapse-free survival in the validation cohort (n=780).

Variables	OS		RFS	
	HR (95% CI)	*p*-value	HR (95% CI)	*p*-value
Age				
>56 years *vs ≤*56 years	1.12 (0.78–1.60)	0.56	0.98 (0.70–1.38)	0.92
Histologic type				
Non-clear cell *vs* clear cell type	0.98 (0.64–1.48)	0.91	0.72 (0.49–1.08)	0.11
Tumor nuclear grade				
1		**<0.001**		**0.001**
2	0.85 (0.44–1.67)		1.14 (0.59–2.19)	
3	1.11 (0.53–2.33)		1.79 (0.89–3.64)	
4	2.98 (1.41–6.29)		2.91 (1.40–6.06)	
Initial TNM stage				
I		**<0.001**		**<0.001**
II	2.18 (1.12–4.22)		2.40 (1.55–3.73)	
III	2.73 (1.72–4.35)		2.60 (1.45–4.67)	
IV	6.58 (3.66–11.83)		11.60 (6.72–20.02)	
Tumor size				
<4 cm		**0.004**		**0.001**
4–7 cm	1.28 (0.79–2.06)		1.39 (0.90–2.15)	
>7cm	2.42 (1.40–4.18)		2.39 (1.43–4.01)	
Pseudocapsule				
Grade 0		**<0.001**		**<0.001**
Grade 1	1.79 (1.07–2.99)		1.80 (1.12–2.89)	
Grade 2	2.64 (1.55–4.49)		2.92 (1.81–4.71)	
Tumor necrosis				
Present vs Absent	1.69 (1.09–2.60)	**0.02**	1.87 (1.25–2.80)	**0.002**
Sarcomatoid transformation				
Present vs Absent	2.72 (1.45–5.08)	**0.002**	2.35 (1.28–4.29)	**0.01**
MI				
Present vs Absent	1.89 (1.23–2.92)	**0.004**	2.02 (1.35–3.04)	**0.001**
ECOG				
1 *vs* 0	2.14 (1.42–3.21)	**<0.001**	1.31 (0.89–1.95)	0.18
PPS				
Low		**<0.001**		**<0.001**
Intermediate	2.53 (1.26–5.11)		3.70 (1.81–7.56)	
High	18.10 (9.35–35.03)		22.09 (11.09–43.99)	

CI, confidence interval; ECOG, Eastern Cooperative Oncology Group; OS, overall survival; RFS, relapse free survival; MI, microvascular infiltration; PPS, pseudocapsule-pathological parameter score. The bold p-values indicate a value less than 0.05, indicating a statistically significant difference.

For RFS in the training cohort (**Table 2**), independent predictors included pseudocapsule grade 2 (HR 2.87, 95% CI 1.72–4.81, *p* < 0.001), pseudocapsule grade 1 (HR 2.28, 95% CI 1.43–3.64, *p* < 0.001), tumor nuclear grade 4 (HR 6.71, 95% CI 2.72–16.56, *p* < 0.001), tumor necrosis (HR 2.19, 95% CI 1.52–3.12, *p* < 0.001), sarcomatoid transformation (HR 2.83, 95% CI 1.61–4.97, *p* < 0.001), microvascular infiltration (HR 1.73, 95% CI 1.15–2.62, *p* = 0.01), and ECOG performance status (HR 1.80, 95% CI 1.25–2.59, *p* = 0.001).

In the validation cohort (**Table 3**), independent predictors of RFS included pseudocapsule grade 2 (HR 2.92, 95% CI 1.81–4.71, *p* < 0.001), pseudocapsule grade 1 (HR 1.80, 95% CI 1.12–2.89, *p* = 0.01), tumor nuclear grade 4 (HR 2.91, 95% CI 1.40–6.06, *p* = 0.004), tumor necrosis (HR 1.87, 95% CI 1.25–2.80, *p* = 0.002), sarcomatoid transformation (HR 2.35, 95% CI 1.28–4.29, *p* = 0.01), and microvascular infiltration (HR 2.02, 95% CI 1.35–3.04, *p* = 0.001).

### 3.3 Construction of the PPS system

The PPS system was developed based on the results of the univariable and multivariable analyses. The scoring system incorporated pathological type (ccRCC, 1 point; non-clear cell renal cell carcinoma [nccRCC], 2 points); Fuhrman grade (1–4 points according to grade); nuclear necrosis (absent, 0 points; present, 1 point); sarcomatoid transformation (absent, 0 points; present, 1 point); microvascular infiltration (absent, 0 points; present, 1 point); ECOG performance status score (0 or 1); TNM stage (1–4 points according to stage); and pseudocapsule status (0–2 points according to grade).

The total PPS ranged from 3 to 16 points, and was categorized into the following three levels: low (3–6 points, 455 cases), intermediate (7–10 points, 695 cases), and high (11–16 points, 410 cases).

### 3.4 Prognostic significance of the PPS system

OS was strongly associated with the PPS system. Kaplan–Meier curve analysis demonstrated worse prognosis in the high-PPS group. The median OS was 63 months and 79.5 months for patients in the high-PPS group of the training and validation cohorts, respectively. In contrast, the median OS was not reached in the low-PPS group (*p* < 0.001) (**Figure 2 C, D**).

**Figure 3:**
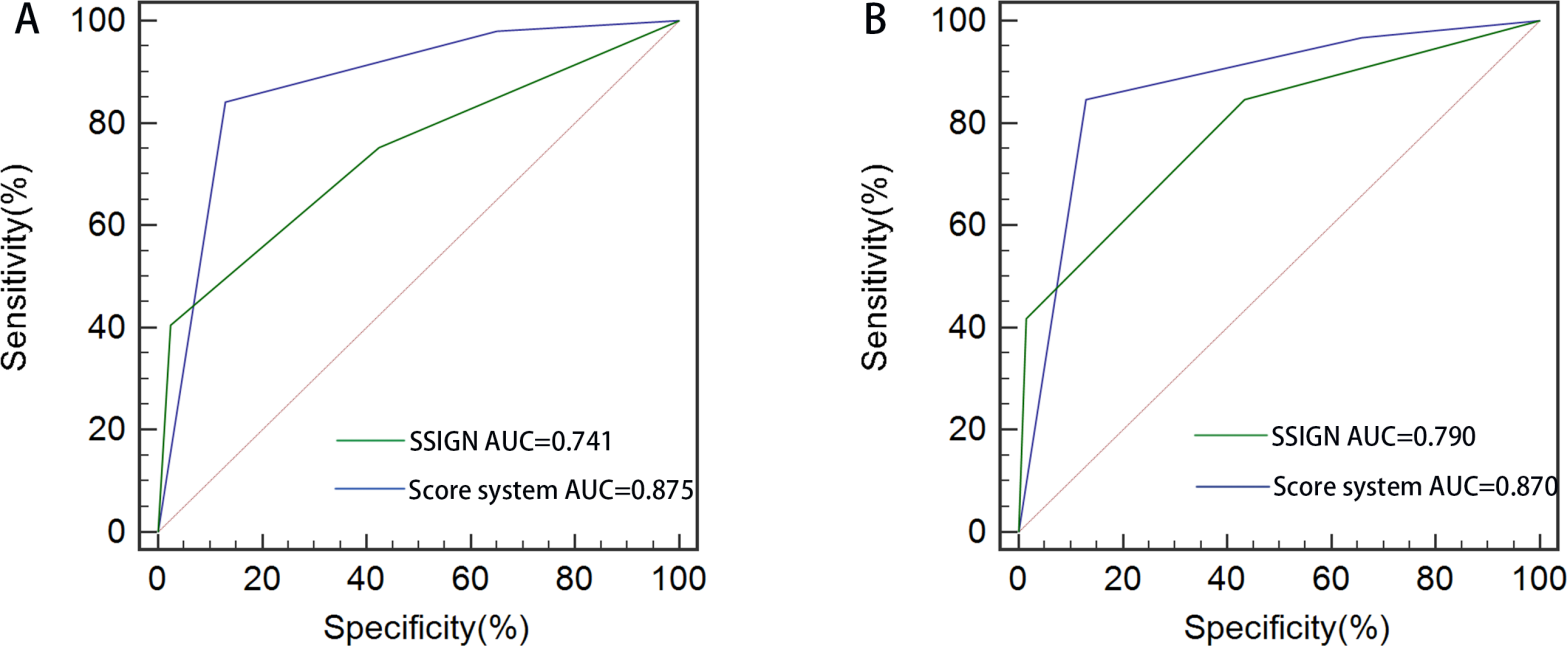
Receiver operating characteristic curve analysis of the predictive accuracy of the Stage, Size, Grade, and Necrosis score and the PPS for 5-year OS. **(A)** Training cohort; **(B)** Validation cohort.

The AUC values for 5-year OS predicted by the SSIGN score alone were 0.741 and 0.790 in the training and validation cohorts, respectively. In comparison, the PPS demonstrated higher predictive accuracy, with AUC values of 0.875 and 0.870, in the training and validation cohorts, respectively (**Figure 3**).

Similarly, the AUC values for 5-year OS predicted by the TNM staging system alone were 0.758 and 0.817 in the training and validation cohorts, respectively. In comparison, the PPS showed higher predictive accuracy, with AUC values of 0.898 and 0.876, in the training and validation cohorts, respectively (**Supplementary Figure**).

Overall, these findings demonstrate the advantages of the PPS scoring system in predicting postoperative survival in patients with RCC.

## 4 Discussion

Tumor infiltration or rupture of the pseudocapsule reflects the growth characteristics of RCC. Previously, tumors confined within an intact pseudocapsule were generally considered to exhibit expansive growth without specific pathological significance. However, through microscopic analysis, we found immune-cell and tumor-cell infiltration around the pseudocapsule. This infiltration affects both the tumor microenvironment and the pseudocapsule, even causing some cell matrices to form a physical barrier that influences tumor invasion and growth. Consequently, the prognostic value of pseudocapsule status has received increasing attention in recent years.

While previous studies have described pseudocapsules in renal tumors, few have investigated the relationship between pseudocapsule status and patient survival (10). Minervini et al. reported that pseudocapsule invasion and breakthrough caused by tumor cell infiltration may affect patient prognosis (11). Snarskis et al. demonstrated that pseudocapsule invasion is related to local recurrence and disease-specific survival (12). Xi et al. showed that pseudocapsule status is a favorable prognostic indicator in RCC and mRCC (13, 14). The results of the present study also confirmed the close association between pseudocapsule status and patient prognosis, with consistent conclusions between the training and validation cohorts. Patients with an intact pseudocapsule showed the best prognosis, whereas those with pseudocapsule breakthrough or no identifiable pseudocapsule showed the worst outcomes (p<0.0001).

For localized RCC, regardless of partial or radical nephrectomy, prognosis is mainly based on UISS and SSIGN scores. The UISS includes TNM stage, Fuhrman grade, and ECOG performance status, whereas the SSIGN score includes tumor stage, tumor size, Fuhrman grade, and tumor necrosis. However, both these systems have limitations and do not include anatomical parameters. According to previous studies and the results of the present large-scale study, pseudocapsule status is closely associated with prognosis and is an independent predictor of OS and RFS. Furthermore, multivariable Cox regression analysis in both the training and validation cohorts revealed that patients with pseudocapsule breakthrough had significantly worse OS and RFS than those with an intact pseudocapsule (p<0.001).

The pseudocapsule is a fibrous connective tissue formed by the expansion and compression of normal renal parenchyma during tumor growth (15).It is crucial for surgical tumor removal. Although some studies have suggested that pseudocapsule rupture may affect prognosis, others have shown that positive local margins do not affect overall outcomes. Therefore, larger clinical studies are required to verify these findings. Minervini et al. confirmed that tumor invasion of the pseudocapsule is closely associated with poor prognosis (11). However, the factors contributing to pseudocapsule invasion remain unclear and may be related to tumor size, Fuhrman grade, microvascular infiltration, or nuclear necrosis. Additionally, immune-cell or collagen-fiber infiltration around the pseudocapsule may contribute to tumor infiltration or pseudocapsule Rupture (16). Azhar et al. reported that the tissue immediately adjacent to the tumor undergoes histological alterations, likely due to tumor-induced compressive effects, resulting in more pronounced inflammation, glomerulosclerosis, nephrosclerosis, and arteriosclerosis near the tumor margin (6). These findings suggest that invasion of the pseudocapsule involves both physical effects related to tumor growth and a series of biological effects. Nevertheless, the specific mechanisms require further exploration.

Beyond the histopathological features examined in our study, accumulating evidence highlights the potential value of metabolic and circulating biomarkers for predicting long-term outcomes after cancer surgery. For instance, a recent study focusing on postoperative metabolic parameters demonstrated that certain serum biomarkers were significantly associated with OS and recurrence risk, although the disease investigated differed from RCC (17). That study emphasized the importance of integrating postoperative biological markers, such as glucose-related indices, lipid profiles, or inflammatory factors, into routine follow-up protocols to improve risk stratification. While our PPS relies on pathological features (pseudocapsule status, Fuhrman grade, TNM stage, etc.), the findings of the aforementioned study suggest that metabolic signatures could provide complementary information on tumor aggressiveness and host response. Consequently, integrating anatomical and pathological parameters with dynamic, serum-based biomarkers might further enhance prognostic accuracy and enable more personalized surveillance strategies. Although the specific metabolic markers analyzed in that study were not directly assessed in our cohort, its findings support the broader concept that postoperative biological monitoring, beyond static pathological variables, contributes substantially to long-term outcome prediction in surgical oncology. Future prospective studies should therefore consider incorporating serial measurements of metabolic and inflammatory biomarkers alongside the PPS to capture both tumor-intrinsic and host-related determinants of prognosis.

In our multivariable analyses, pseudocapsule grade 2 was associated with HRs for OS of 3.94 and 2.64 in the training and validation cohorts, respectively. These values are modest and comparable to those of other established predictors such as tumor size and microvascular infiltration. This finding is biologically plausible because pseudocapsule disruption reflects local tumor aggressiveness but is unlikely to be the sole determinant of long-term survival in most patients. Moreover, OS is influenced by multiple factors beyond cancer biology, including patient age, comorbidities, and subsequent therapies.

Based on the results of the multivariable analyses, we combined pseudocapsule status with established pathological parameters to construct the PPS system. The primary finding of our study is not the isolated effect of pseudocapsule status, but the performance of the combined PPS model. When pseudocapsule status was integrated with seven routine pathological parameters (histological subtype, Fuhrman grade, tumor necrosis, sarcomatoid transformation, microvascular infiltration, ECOG performance status, and TNM stage), the resulting PPS demonstrated substantially higher HRs for OS. As shown in **Tables 2**, **3**, the PPS was strongly associated with both OS and RFS in the training and validation cohorts. The effect sizes for the high-PPS group were an order of magnitude larger than those of any single pathological parameter, and the associations for OS and RFS were similar within each cohort. A high PPS reflects the simultaneous presence of multiple adverse factors, such as high Fuhrman grade, advanced TNM stage, microvascular infiltration, sarcomatoid transformation, and pseudocapsule breakthrough, thereby representing a highly aggressive tumor phenotype. Such patients are at increased risk of both local recurrence (reflected by RFS) and cancer-specific mortality, which ultimately drives OS. In contrast, a low PPS indicates indolent tumor behavior, leading to excellent long-term survival.

Thus, the PPS system may provide a clinically useful method for integrating multiple prognostic dimensions, and its strong association with OS likely reflects the combined effect of these adverse pathological characteristics rather than an overinterpretation of pseudocapsule status alone. We propose that the PPS, which incorporates pseudocapsule status as a component, effectively stratifies patients according to their overall tumor aggressiveness, thereby offering robust prediction of both RFS and OS.

Furthermore, the PPS system showed significantly higher AUC values for 5-year OS prediction than the SSIGN score (training cohort: *p* < 0.0001; validation cohort: *p* = 0.0007). These findings indicate that the assessment of pseudocapsule status is crucial in the postoperative pathological assessment of patients with RCC, providing an important reference value for prognosis evaluation. H&E staining is already routinely used for evaluation. Thus, combining routine pathological indicators with the PPS system can more accurately predict patient prognosis without increasing evaluation costs.

This study has several limitations. First, its retrospective design may have introduced inherent sources of bias, although all pathological slides were prospectively reevaluated by experienced pathologists.

Second, to comprehensively evaluate the pseudocapsule status in RCC, we included a diverse patient population, including those who underwent partial or radical nephrectomy, had nccRCC, received tyrosine kinase inhibitors (TKIs), and underwent postoperative immunotherapy. For patients with partial recurrence, treatment options included TKIs such as sunitinib or pazopanib, and PD-1 monoclonal antibodies for immunotherapy. Such heterogeneity may have influenced patient outcomes. To minimize the potential impact, we separated the patients into training and validation cohorts, which yielded comparable results.

Third, the study period spanned several years (2007–2012), during which surgical techniques, pathological assessment standards, and systemic therapies evolved. These changes include increased use of minimally invasive partial nephrectomy, updates to ISUP grading, and wider adoption of TKIs and immune checkpoint inhibitors. This temporal heterogeneity may have influenced patient management and outcomes, and therefore represents a potential source of unmeasured bias.

Fourth, the SSIGN score is a prognostic system developed specifically for ccRCC; however, our cohort included some patients with nccRCC, which may have also introduced bias. Future studies should aim to conduct more precise assessments and prognostic predictions specifically for ccRCC.

Fifth, although the PPS demonstrated improved prognostic performance compared with existing systems, the PPS scoring system was developed and internally validated within a single institutional cohort, which carries a potential risk of overfitting. Consequently, its predictive performance may be overestimated when applied to external populations. External validation in independent, multicenter cohorts is therefore essential before the PPS can be recommended for routine clinical use.

Finally, an additional limitation is that the pseudocapsules were classified according to status alone, without accounting for their thickness. Future studies should investigate potential associations between pseudocapsule thickness and clinicopathological parameters.

In conclusion, our findings demonstrate the close relationship between pseudocapsule status and patient prognosis. By combining pseudocapsule status with established pathological parameters, the PPS system may better predict patient prognosis without increasing examination costs. Future studies should validate our findings and confirm the clinical utility of this system.

## Data Availability

The original contributions presented in the study are included in the article/**Supplementary Material**. Further inquiries can be directed to the corresponding authors.
