# Supplementary figures and images for: Pseudocapsule status combined with pathological parameters predicts prognosis in renal cell carcinoma
Source: Front Oncol. 2026 Jun 22;16:1779539. doi: 10.3389/fonc.2026.1779539 (PMC13333514; doi:10.3389/fonc.2026.1779539)

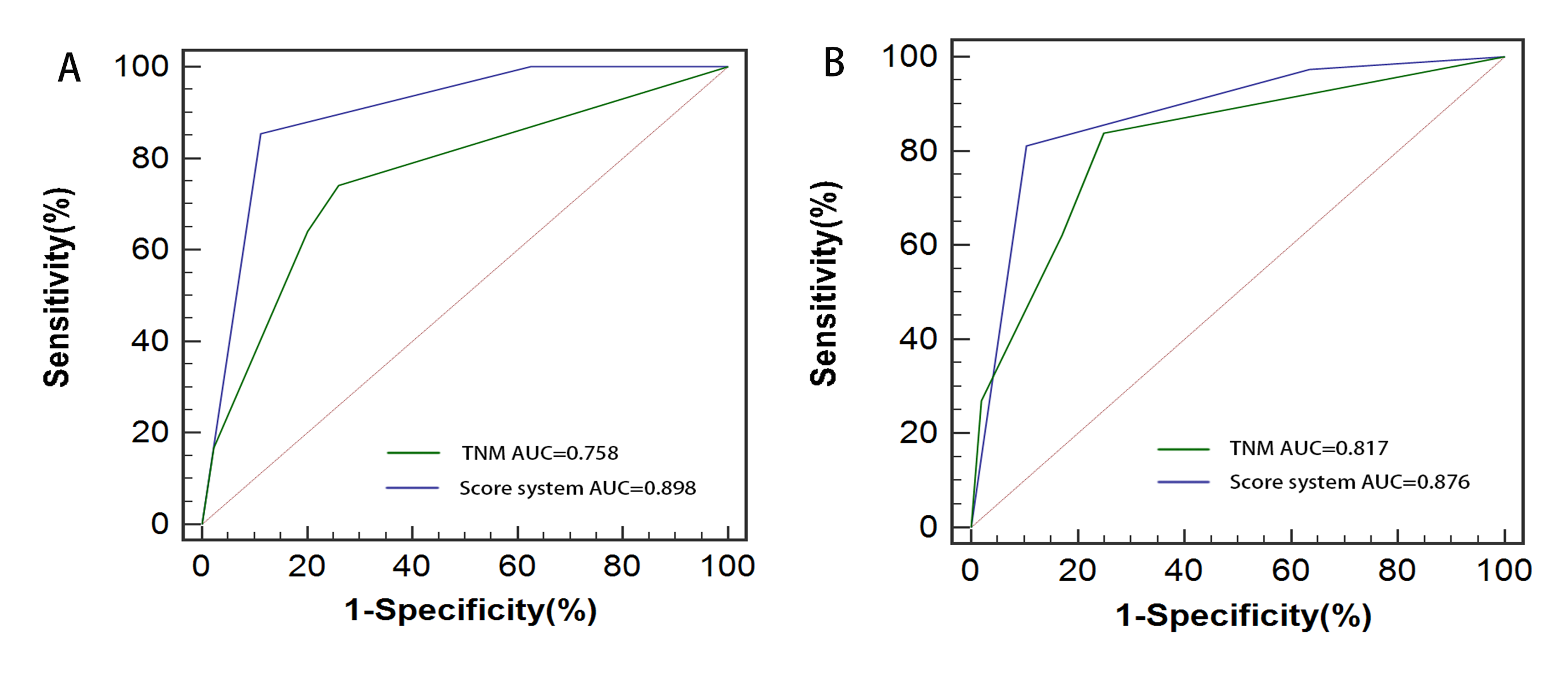

Supplement: Supplementary file 1 [file Image1.tif]
